# Valorisation of Buckwheat By-Product as a Health-Promoting Ingredient Rich in Fibre for the Formulation of Gluten-Free Bread

**DOI:** 10.3390/foods12142781

**Published:** 2023-07-21

**Authors:** Ángel L. Gutiérrez, Marina Villanueva, Daniel Rico, Joanna Harasym, Felicidad Ronda, Ana Belén Martín-Diana, Pedro A. Caballero

**Affiliations:** 1 Food Technology, Department of Agriculture and Forestry Engineering, University of Valladolid, 34004 Palencia, Spain; angelluis.gutierrez@uva.es (Á.L.G.); marina.villanueva@uva.es (M.V.); mfronda@uva.es (F.R.); 2 Agrarian Technological Institute of Castilla and Leon (ITACyL), Ctra. Burgos Km 119, Finca Zamadueñas, 47071 Valladolid, Spain; ricbarda@itacyl.es (D.R.); mardiaan@itacyl.es (A.B.M.-D.); 3 Bio-Ref Lab, Department of Biotechnology and Foods Analysis, Institute of Chemistry and Food Technology, Faculty of Engineering and Economics, Wrocław University of Economics, 53-345 Wrocław, Poland; joanna.harasym@ue.wroc.pl

**Keywords:** buckwheat hull, gluten-free bread, dough rheology, fibre content, antioxidant capacity

## Abstract

Bread is a widely consumed food that has often been used as a vehicle for functional ingredients such as dietary fibre. Fibre-rich breads have beneficial physiological effects on health, helping to combat chronic pathologies such as cardiovascular disease, diabetes, and certain types of colon cancer. The aim of this study is to evaluate the technological and nutritional effects of the inclusion of buckwheat hull particles (BH) at two addition levels (3 and 6%) and two particle sizes (fine, D_50_: 62.7 μm; coarse, D_50_: 307 μm) in a gluten-free (GF) bread formulation. A significant (*p* < 0.05) increase in the dough elastic modulus (G’) was observed for all doughs containing BH, from 712 Pa for a rice-based dough to 1027–3738 Pa for those containing BH. Compared to rice-based breads, those containing BH showed a significant (*p* < 0.05) increase in total dietary fibre content (from three to five times) and in antioxidant capacity (from 78 to 290 mg TE/100 g dw. in the ORAC test). Breads containing fine BH at a level of 3% had similar sensory properties to the rice-based bread, demonstrating that it is possible to improve the TDF content while maintaining the sensory quality of the GF bread.

## 1. Introduction

Dietary fibre (DF) is currently attracting particular interest from consumers and researchers. Many claims have been associated with its intake, from the reduced glycaemic response, increased faecal volume, maintenance of normal blood cholesterol levels to the reduction of certain types of cancer [1]. Institutions such as the European Food Safety Authority (EFSA) recommend a fibre intake of at least of 25 g/day for normal laxation in adults and an intake above this amount for a reduced risk of coronary heart disease, type 2 diabetes and improved weight maintenance [2]. There are many fibre sources in foods, including legumes, fruits, vegetables and whole grains, in which the fibre is found especially in the outer parts of the seeds. DF is mainly composed of non-starch polysaccharides with significant variability in structure and physicochemical properties that influence their physiological response. Existing methods can differentiate fibre types by their solubility, although this characteristic is not always key to predicting their physiological effect [3]. In addition to solubility, other biochemical properties such as viscosity, fermentability and gel-forming ability have been associated with their potential health benefits [1]. While soluble, high viscous and gel-forming fibres such as β-glucans or psyllium may have health benefits in controlling glycaemia and cholesterol, and fibres with low fermentability may have a constipation or laxative effect. In turn, the physiological effects of insoluble fibres depend on their particle size; whereas fine particles can lead to a constipating effect, coarse particles can have a laxative effect [4].

Despite the evidence for the health benefits of DF intake, there is still a lack of DF in the Western diet [5]. Among special needs populations, celiac disease patients (approximately 1% of the Western population) are particularly susceptible to DF deficiency, due to a general imbalance in the nutritional profile of specific gluten-free (GF) foods. This imbalance has been attributed to an increase in fat intake that displaced the consumption of fibre and complex carbohydrates [6]. Different authors have also found that coeliac diets tend to be higher in fat and sugar and lower in fibre, proteins, micronutrients and bioactive compounds than non-coeliac diets [6,7,8].

Public institutions recommend increasing DF intake through dietary guidelines that emphasise the importance of choosing a variety of foods, including concentrated sources of fibre [9]. According to EU Regulation No 1924/2006 in EU, fibre-containing products can be specifically labelled for the use of the terms “source of fibre” and “high in fibre” as health claims if the product contains at least 3 and 6 g of fibre per 100 g, respectively, that demonstrate a beneficial physiological effect [2]. Strategies that can also help to meet DF consumption recommendations include adding small amounts of non-soluble fibre to foods [5] or the regular consumption of whole grains, as they are rich in fibre and other biologically active compounds, including vitamins, minerals and phenolics [10].

The innovation and development of acceptable-tasting fibre-enriched food products could be considered as a feasible strategy to encourage consumers to increase their intake of fibre. Bread can be considered as a suitable vehicle for fibre-enriched products, as it is a versatile and affordable staple food with balanced nutritional properties. The addition of DF to refined-flour-based breads can improve their nutritional profile, also providing micronutrients and bioactive compounds with antioxidant capacity such as flavonoids, carotenoids and polyphenols [10,11]. However, depending on the fibre type, the dosage and the particle size, the incorporation of DF in breadmaking has a greater or lesser effect on the rheological properties of the dough and the quality of the final product [12,13]. This is even more important in the production of GF breads, as the lack of gluten affects the viscoelastic properties of dough and hinders the gas retention of breads during proofing, resulting in poor quality breads [14].

Traditionally, wholemeal flours have been used for fibre enrichment in breads; however, because of their potential to broaden the health benefits, research into the addition of other DF sources is of growing interest [15]. Among these novel sources of DF, buckwheat (BW) is considered a raw material of great interest for the production of functional foods. In addition to its contribution to dietary fibre, this pseudocereal contains polyphenols in the form of flavonoids, which have a high antioxidant capacity [16] and other physiological effects such as the ability to lower blood pressure [15]. Among the parts of BW grains, dietary fibre is remarkably abundant in the outer layers of the seed, in the seed coat or the pericarp (hull). The latter plant material consists mainly of cellulose, lignin and non-cellulosic polysaccharides and contributes mainly to the insoluble fraction of DF [17]. Phenolic compounds are also mainly present in the BW hull [18], which is also a good dietary source of minerals [17]. The hulls are obtained during the processing of buckwheat kernels in roller or impact mills [17]. Although this raw material is generally considered as industrial waste or a by-product [19], several industrial uses for BW hulls have been proposed, such as pillow fillers, packaging containers for food cans and as a source of potash and natural dyes [20]. The potential of this by-product as a functional ingredient, for its dietary fibre content, could also contribute to waste reduction and indirect income generation [21]. Previous studies confirmed the possibility of obtaining extracts from these plant wastes, contributing to sustainability by promoting a ‘circular economy’ [22]. However, there are very few studies on the use of BW hull as an ingredient in food processing. Recently, its application in the production of wheat bread to reduce product staling has been reported [23]. However, their use in GF bakery products, which are characterised with a starch-dominated matrix in which hydrocolloids play an important role in ensuring gas retention during the baking process, has not yet been proposed.

Similar to flours and other fibre sources, the degree of comminution of the BW hull particles is a key factor in their nutritional and functional value. It has been reported that BW hull micronization treatment could exert positive effects on bioactive and functional properties, enhancing its potential application in functional foods [24]. From a technological point of view, it is of interest to know how the inclusion of buckwheat hull particles (BH) of different sizes in complex matrices such as GF dough and bread could affect their nutritional properties and organoleptic and textural quality.

Therefore, the aim of this study is to evaluate the use of this by-product as a functional ingredient in a GF bread formula, assessing its impact on the rheological properties of the dough and the quality of the resulting GF bakery product. For this purpose, two batches of BH particles (one corresponding to a fine BH particle size and the other to a coarser one) and two addition percentages were studied in order to obtain at least 3 and 6% (*w*/*w*) of DF in the final product.

## 2. Materials and Methods

### 2.1. Raw Materials

Rice flour was provided by Herba Ricemills S.L.U (89.77% carbohydrates, 1.15% of dietary fibre, 9.04% protein, 0.39% ash and 0.81% fat in dry weight basis). Salt, sugar and sunflower oil were acquired from a local supermarket. The rest of the ingredients were as follows: HPMC (Methocel K4M, Dow Wolff Cellulosics, Midland, TX, USA) as well as water and dried yeast (European, Istanbul, Turkey). Split pieces of buckwheat (BW) hulls (BH) were obtained from two commercial Polish products (Sante, Warsaw, Poland): a coarse BW hull batch (CBH; D_50_: 307 μm) and a fine BW hull batch (FBH; D_50_: 62.7 μm). The proximate composition of both ingredients were as follows: 90.80% carbohydrates (87.34% dietary fibre), 6.22% protein, 2.23% ash and 0.75% fat for CBH as well as 93.78% carbohydrates (89.71% dietary fibre), 4.21% protein, 1.93% ash and 0.08% fat in dry weight basis (dw) for FBH.

### 2.2. Dough Formulation and Breadmaking Procedure

Dough samples were using the following gluten-free (GF) formula on the basis of 100 g of rice flour (13% moisture content): water (110%), sunflower oil (6%), sugar (5%), HPMC (2%) and salt (1.5%). Given the dietary fibre content of each BH sample and based on 100 g of rice flour, the BH added was the required amount to obtain at least 3% and 6% of fibre content coming from BH in order to comply with EU Regulation No. 1924/2006 on the health claims “source of fibre” and “high fibre”, respectively. The amounts of BH on a wet basis added to the formulation were 3.61% and 3.83% of CBH and FBH, respectively, for the dough and bread samples CB3 and FB3, respectively, and 7.23% and 7.66% of CBH and FBH, respectively, for the samples CB6 and FB6. The dough preparation and the breadmaking process were performed following the procedure of Villanueva et al. [25]. Briefly, after mixing the solid and liquid ingredients for 8 min with a professional mixer (Model 5KPM50, KitchenAid, St. Joseph, MI, USA) at speed 4, the dough samples were placed into aluminium pans for proofing (28 °C and 85% relative humidity) for 50 min. After that, the pans were distributed for baking (40 min at 190 °C) in an oven (EMD-Salva, Salva, Lezo, Spain). Finally, bread samples were allowed to cool down for 1 h before carrying out analysis. One bread of each formulation was stored at 4 ± 2 °C in polyethylene bags in order to study the effect on staling at seven days.

### 2.3. Rheological Properties of Doughs

An oscillatory test was performed with a Kinexus Pro+ rheometer (Malvern Instruments, Malvern, UK) using parallel plate geometry (40 mm diameter) with a serrated surface and a gap of 1 mm. Following the procedure of Villanueva et al. [25], frequency sweeps were carried out from 10 to 0.1 Hz in the linear viscoelastic region (LVR) once it was detected in a previous stress sweep carried out from 0.1 to 500 Pa. The stress value selected to perform the frequency sweeps was fixed at 0.5 Pa. Maximum shear stress values (τ_max_) at which the dough’s structure started to degrade were determined at 10% below the LVR stress value. Elastic and viscous moduli (G′ and G″) and loss tangent (tan δ) from output frequency sweep data were fitted to the power law model, and the following coefficients were obtained: G′_1_ and G″_1_ represent the elastic and viscous coefficients at a frequency of 1 Hz, and the “a”, “b” and “c” exponents quantify the frequency dependence of the elastic and viscous moduli and the loss tangent, respectively. The complex modulus (G*_1_ = {(G′_1_)^2^ + (G″_1_)^2^}^1/2^) and the stress at the crosspoint (G′ = G″) were also determined. All tests were carried out in duplicate.

### 2.4. Proximal Composition of Bread Samples

Proximal analysis was performed by measuring carbohydrate, protein, fat, moisture and ash of the bread samples. The nitrogen content was determined with the Dumas method (AOAC 2005, method 990.03) [26] in an elemental analyser (Leco Corp., St. Joseph, MI, USA). A conversion factor of 6.25 was used to obtain the protein content. Total fat was assessed using a fat extracting unit (Soxtec System 2055 Tecator, Foss, Hillerød, Denmark) using dried extracts obtained from bread samples with petroleum ether (BP 40–60 °C) (AOAC 2005, method 2003.05) [26]. Ash content was determined by incineration of the samples in a muffle furnace at 550 °C during 24 h (AOAC 2005, method 923.03) [26]. Finally, carbohydrates were calculated with difference. The total dietary fibre (TDF) concentration was obtained using a kit (Sigma, St. Louis, MO, USA) and the manufacturer’s instructions based on AOAC method 985.29 [26]. Results were expressed in dry weight basis (dw). All measurements were performed in duplicate.

### 2.5. Total Phenol Content (TPC) and Total Antioxidant Capacity (TAC)

Total phenol content (TPC) method with Folin–Ciocalteu reagent was considered, as grains are rich in phenolic compounds, accounting for most of their antioxidant activity. The extraction used was previously optimised for the soluble fraction of these types of matrices [27]. Results were expressed as mg of gallic acid equivalents (GAE) per 100 g of sample on a dry weight basis (dw). Samples were evaluated in duplicate.

On the other hand, total antioxidant capacity (TAC) of the extracts was also evaluated through spectrophotometric- and fluorescence-based methods. In order to evaluate a wider range of antioxidants, different methodologies involving the two main mechanisms for radical scavenging, single electron transfer (SET) and hydrogen atom transfer (HAT) reactions, were used. The TAC of the samples was measured on extracts and directly on grounded bread samples (Quencher, Q-) using DPPH• and ABTS•+ radicals, following the procedure of Rico et al. [28]. In addition, the oxygen radical absorbance capacity (ORAC) and the ferric reducing ability potential (FRAP) assays were also performed following the methodology described by Rico et al. [29]. All absorbance data were recorded with a microplate reader (Fluostar Omega, BMG) and results were expressed in dry weight basis (dw) as mg Trolox equivalent (TE) per 100 g of sample (DPPH, Q-DPPH, ABTS, Q-ABTS and ORAC) and as mmol Fe^2+^/100 g (FRAP).

### 2.6. Bread Microstructure

The microstructure of ground bread samples was analysed by scanning electron microscopy (SEM) using a Quanta 200-F (FEI, Hillsboro, OR, USA) at high vacuum conditions using a secondary electron detector. Prior to analysis, each sample was coated with gold. Microphotographs of the metallised samples were acquired at an accelerating voltage of 15 keV at magnifications of 500×, 1000× and 3000×.

### 2.7. Bread Quality Evaluation

The bread samples, once cooled for 1 h and removed from the moulds, were weighed to calculate the baking loss. The bread volume was measured using a VolScan Profiler 300 (Stable Micro Systems, Godalming, UK) and the loaf specific volume was determined by dividing the volume of the product obtained by the weight of the bread [30].

Bread crumb texture was assessed with a TA-XT2 Texture Analyzer (Stable Micro Systems, Surrey, UK) and the software “Texture Expert” (V.2.63), following the procedure of Villanueva et al. [25].

Crumb grain characteristics of breads were assessed using digital image analysis. Images of bread slices were previously acquired at 600 dots per inch with an Hp Scanjet G3110 scanner (Hewlett Packard Enterprise, Palo Alto, CA, USA). The analysis was performed on a 30 × 50 mm square taken from the centre of the slice. Images were processed using ImageJ 1.51 j8 software (Wayne Rasband, Bethesda, MD, USA). The parameters obtained were mean cell area (mm^2^), cell density (cells/cm^2^), void fraction (percentage of the cross-sectional area of the crumb occupied by detected cells) and crumb grain uniformity (ratio of the number of small to large cells).

Crust and crumb colour was determined using a Minolta colorimeter (CN-508i, Minolta, Co., Ltd., Tokyo, Japan), following the procedure used by Villanueva et al. [25]. Colour differences of each buckwheat-hull-containing bread (BHB) with respect to the control were obtained using the equation: ΔE = {(ΔL*)^2^ + (Δa*)^2^ + (Δb*)^2^}^1/2^.

### 2.8. Sensory Evaluation

A descriptive sensory test was conducted on control and fibre-enriched breads. A total of 54 panellists (aged 25–55) rated several attributes for each sample using a nine-point hedonic scale, ranging from 1 (extremely dislike) to 9 (extremely like). The breads were assessed on the basis of crumb and crust colour, flavour, crumb texture, taste, aftertaste and overall acceptability.

### 2.9. Statistical Analysis

Using Fisher´s Least Significant Difference (LSD) test to describe means with 95% confidence, one factor (ANOVA) analysis of variance was performed to assess significant differences between samples. A multifactorial analysis of variance was also carried out to provide information about the individual factors and the interactions between them. The two factors studied were the particle size of the BH added to the dough and its addition level. The Statgraphics Centurion XVII program (Statsoft Inc., St. Tulsa, OK, USA) was used for statistical analysis.

## 3. Results and Discussion

### 3.1. Rheological Properties of Doughs

The effects of the buckwheat hull particles (BH) addition at different particle sizes (fine, F- and coarse, C-) and at two addition levels in the gluten-free (GF) doughs were evaluated using dynamic oscillatory tests. The results presented in Table 1 showed that both factors and their interaction had a significant effect (*p* < 0.01) on the viscoelastic properties of the dough except for τ_max_. A significant increase (*p* < 0.05) in the elastic (G′) and viscous (G″) moduli was observed for all BH-containing doughs compared to the control. The G′_1_ values of the fine BH (FBH)-containing doughs increased by two and five times at addition levels of 3% and 6%, respectively, whereas a smaller increase was observed for the coarse BH (CBH)-containing doughs. Similarly, the G″_1_ values of the dough containing 6% FBH increased by three and a half times compared to the control dough. These results led to a significant decrease (*p* < 0.05) in the loss tangent for the doughs containing BH compared to the control, indicating a more elastic behaviour.

Previous studies conducted in gluten-free (GF) systems have reported that the addition of insoluble fibre particles led to an increase in dough firmness and consistency [31,32]. As it was reported by Sciarini et al. [31], dough viscosity is related to the high water holding capacity (WHC) of fibres. Furthermore, Zhu et al. [24] showed that the finer the BH particle, the more WHC it had. Wang et al. [23] verified higher water absorption of wheat doughs enriched with cell-scale BH (50–10 µm) than those enriched with tissue-scale BH (500–100 µm). Thus, the higher viscoelastic moduli for FBH-containing doughs compared to the coarse ones, indicating a higher dough consistency [33], could be explained by variations in the WHC of the different BH fractions used in this study.

The dependence of the viscoelastic moduli with the frequency, measured with the exponents “a” and “b”, also followed a similar trend. All fibre-enriched doughs had significantly (*p* < 0.05) lower values for these parameters than the control, with the greatest difference corresponding to the 6% FBH dough, indicating that the addition of BH improved the structural stability of the dough, particularly for the FBH doughs.

The maximum stress value (τ_max_) that the dough can withstand before the elastic behaviour begins to decline showed a significant (*p* < 0.05) increase with the addition of 6% FBH compared to the control. In turn, the crossover point, or the stress level at which the elastic behaviour of the dough changes to a viscous one, revealed significant differences (*p* < 0.05) in all BH-supplemented doughs compared to the control. Following the same trend as the elastic modulus, the 6% FBH dough showed an increase of three and a half times the value observed for the control. The increase in dough mechanical strength with the addition of insoluble fibres has previously been observed by C. Wang et al. [34], who attributed the internal rigidity of the dispersed fibres in the dough for the observed increase in dough resistance to kneading.

### 3.2. Proximal Composition of Bread Samples

Table 2 shows the nutritional composition of breads. As expected, the addition level of BH was significant (*p* < 0.01) for total dietary fibre (TDF). The TDF content in bread increased significantly (*p* < 0.05) with the increasing BH addition. The lowest TDF values corresponded to the control sample, followed by breads with a 3% hull addition (the GF bread containing the fine and coarse BH–FB3 and CB3 samples, respectively), while the highest TDF content was found for those breads at 6% of addition level of CBH and FBH (CB6 and FB6 samples, respectively). The addition of BH, regardless of particle size, increased the TDF content of GF breads (GFB) from three to five times more than that of the control samples. Phimolsiripol et al. [35] observed a significant increase in the TDF of a rice-based GFB with the addition of 10% defatted rice bran flour as a fibre-enriching ingredient, from 2.29% in the control bread to 5.97% for bran-enriched bread. In this research, the contribution to TDF of rice flour was minimal (its dietary fibre content was 1.15% *w*/*w*, dry basis), so several factors could have played an important role in increasing the DF content of buckwheat-hull-containing breads (BHBs). As it was expected, the most important factor was the addition of BH in the GF bread formula. However, the amount of HPMC added, which is considered a fibre [14], might also have contributed to that increase in the TDF content. The value obtained showed that it could be possible to obtain the “high fibre” claim (EU Regulation No 1924/2006) [2] even with BHBs formulated with the lowest BH addition.

With regard to the other components analysed (fat, protein and ash), no significant (*p* < 0.05) differences were observed. The low presence of these components in BH, together with the relatively low addition level of BH in the GFB formula, would explain the lack of significant differences in the breads tested.

### 3.3. Total Phenol Content (TPC) and Total Antioxidant Capacity (TAC)

Total phenol content (TPC) and total antioxidant capacity (TAC) were evaluated in all GFB samples (Table 3). TPC was significantly (*p* < 0.01) affected by the BH addition level and ranged from 0.3 to 2.1 mg GAE/100 g. Significantly (*p* < 0.05) higher TPC values were also found for BHBs enriched at 6% compared to those enriched at 3%. These are expected results as a high phenol content was reported in the BW hull [36,37]. The particle size also showed a significant (*p* < 0.05) effect on TPC. Regardless of the concentration of BH used, higher TPC was observed in those breads enriched with smaller particle size. This is in line with the findings reported by Zhu et al., who reported a significant (*p* < 0.05) higher phenol content for BH particles after undergoing an ultrafine grinding treatment [24].

With regard to antioxidant parameters, the results obtained from the extracts showed a significant (*p* < 0.05) increment in antioxidant activity with increasing BH addition. Additionally, in the ORAC and FRAP tests, the effect of particle size was only significant (*p* < 0.05) in bread enriched at 6%, where bread enriched with CBH had significantly (*p* < 0.05) lower antioxidant activity compared to its FB’s counterpart. However, when the antioxidant activity was evaluated directly on the samples (Q-DPPH and Q-ABTS tests) the hull particle size showed a significant (*p* < 0.01) effect, probably due to the non-soluble phenolic compounds associated with fibre. This effect was also previously reported by Zhu et al. [24], who suggested that the grinding could cause damage to the fibre matrix, favouring the extractability of phenolic compounds, which are mainly responsible for the antioxidant activity. Although the use of BH to improve the antioxidant capacity of bread has already been proposed [38], the literature on this application of this BW by-product is still scarce. However, the use of other edible parts of the BW grain is more widespread [39]. A previous study showed higher TPC and TAC values for breads containing buckwheat flour [40]. The observed increases in antiradical activity could be attributed to the release of antioxidant compounds from cell wall degradation during dough fermentation and also to the Maillard reaction products associated with the baking process.

### 3.4. Bread Microstructure

**Figure 1:**
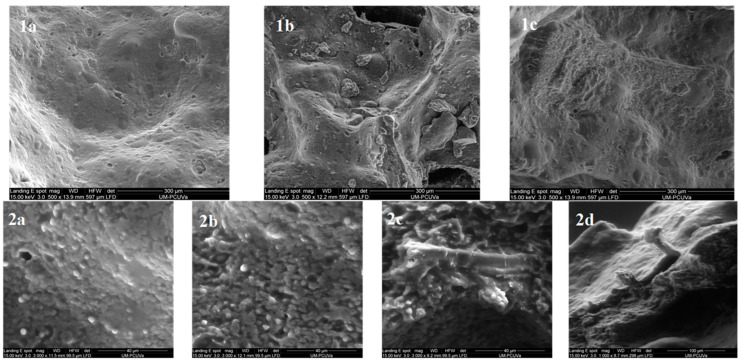
Scanning electron microscope images of the bread crumbs. (**1a**,**2a**): control bread sample; (**1b**,**2b**): bread containing coarse buckwheat hull particles at an addition level of 3%; (**1c**,**2c**,**2d**): breads containing coarse buckwheat hull particles at an addition level of 6%.

Figure 1 shows the photomicrographs of the different breads obtained by BH fortification. Compared to the uniform and smooth structure of the control crumb (Figure 1(1a,2a), the crumb of the BHBs showed a more irregular surface, with heterogeneous structures that exposed some starch granules (Figure 1(1b,1c,2b,2c)). BH particles are mainly composed of non-starch polysaccharides such as hemicellulose, a typical component of cell walls in dicotyledonous plants [41] and lignin [17]. Due to their brittle nature, the BW dehulling process could promote an irregular, sharp and sheet-like appearance of BH particles, which would contribute to the irregular structure observed in the crumb of BH-enriched breads. In contrast, the starch granules in these samples were more regular and well-defined. The preservation of the granular starch structure could be associated with the lower availability of water in the medium, as the hull particles would exhibit a high water retention capacity [24] and would compete with starch for water.

HPMC is a well-known hydrocolloid used to provide a network structure in gluten-free systems, helping to stabilise gas cell expansion [14] and develop a continuous matrix [32]. As can be seen in Figure 1(2c,2d), hull particles stood out from the crumb structure, which could have hindered the HPMC network, leading to air bubble collapse. Previous studies proved that insoluble fibre could promote gas cell interference, destabilising the aerated structure of the dough [42]. The results obtained in this study showed that this effect was highly dependent on the size and the level of BH addition, with a stronger effect when using a higher BH particle size and a higher BH concentration.

### 3.5. Bread Quality Evaluation

The BH particle size and its interaction with the BH addition level showed a significant effect (*p* < 0.01 and *p* < 0.05, respectively) on the bread volume (Table 4). The use of CBH at both addition levels resulted in a significant (*p* < 0.05) reduction in the bread volume and specific volume, with a significant (*p* < 0.05) reduction of 17% in bread specific volume for CB3 samples compared to the control bread. In contrast, no significant differences were found for FBs at any level of addition compared to the control bread. As compacted bread could be perceived in an undesirable way by consumers [43], the use of FBH in GFB formulations at the doses tested in this study could be used for appropriate GFB formulation. The reduction in bread volume of GFBs as a result of the addition of large and insoluble fibre particles was previously reported by Martínez et al. [42], who suggested that these particles interacted with the internal structure of the dough and promoted a reduced gas retention capacity. Conversely, the addition of BH particles to wheat bread affected bread specific volume when cell-scale BH (50–10 µm) was added instead of the coarser tissue-scale BH (500–100 µm) [23].

The addition of BH particles in the bread formulation had a remarkable effect on the weight loss of GFBs as both factors, the BH particle size and BH addition level, were significant (*p* < 0.05 and *p* < 0.01, respectively). According to the ANOVA results, significantly (*p* < 0.05) lower values of weight loss were found for all BHBs compared to the control. Among BHBs, the FB3 sample had the lowest percentage of weight loss. These results were similar to those obtained by Zanoletti et al. [44], who previously reported that ingredients with water retention sites, such as buckwheat bran particles, have the ability to increase the moisture content of wheat dough and reduce the baking loss of breads. Complementarily, Zhu et al. [24] showed that the reduction of hull particle size increased the water holding capacity of these fibres, which would reduce the weight loss of bread during baking.

The level of BH addition showed a significant (*p* < 0.01) effect on bread crumb hardness. All BHBs exhibited significantly (*p* < 0.05) higher hardness values (*p* < 0.05) than the control, with the exception of the FB3 sample. The observed increase was particularly noticeable for the CB samples as the hardness increased from 1.8 N for the control to 3.2 N and 3.1 N for the CB3 and CB6 samples, respectively. A significant increase in crumb hardness with increasing levels of insoluble fibres in GFB has been previously reported [31]. Martínez et al. [42] also reported a significant (*p* < 0.05) increase in hardness when using coarse fibres instead of finer ones. Increased crumb firmness in bread formulations after the addition of buckwheat fibre was also previously observed in the study of Zanoletti et al. [44], where fine and coarse buckwheat bran particles at different doses enriched a wheat-based bread.

All breads analysed showed an increase in crumb hardness after 7 days, but the FB3 sample exhibited the lowest value, followed by the control bread and the FB6 sample. Higher and significant (*p* < 0.05) crumb hardness values were observed for the CB samples compared to the control and regardless of the addition level. Increase in firmness during storage was related to the water retention capacity of the fibre, as Sciarini et al. [31] observed. Those authors indicated that the addition of fibre reduced water loss during storage, resulting in the hardening rate of the crumb to slow down.

A significant effect of the BH addition level factor was found regarding crumb springiness and chewiness (*p* < 0.05 and *p* < 0.01, respectively), although any statistical difference was observed for the BHBs with respect to the control.

Conversely, the effects of the BH addition level, BH particle size and the interaction between the two factors were found to be significant for cohesiveness. CBs showed significantly (*p* < 0.05) lower crumb cohesiveness than the control bread, but this detrimental effect was not found in FBs. The reduction in crumb cohesiveness with the addition of buckwheat bran particles was also reported by Zanoletti et al. [44] in a wheat bread formulation. This reduction was attributed to the appearance of micro-fractures in the air cell walls during the crushing. Micro-fractures could be promoted by the inclusion of coarse solid particles, which would promote the apparition of discontinuities in the crumb network. This hypothesis was consistent with the changes in crumb microstructure observed in this study (Figure 1(2d)), as CBH could become intertwined with the polymeric crumb network, thus promoting the appearance of micro-fractures. The resilience of the breads, which is related to the crumb recovery ability after a compression cycle, followed a similar trend to the cohesiveness. However, in this study, the effect of the BH addition level was not found to be significant.

**Figure 2:**
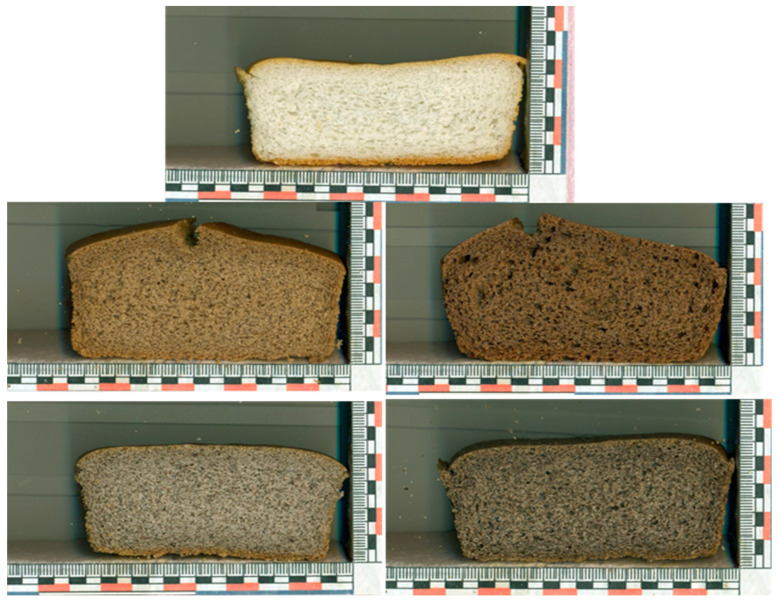
Images of the internal crumb structure of gluten-free breads. C: Control bread. FB3/CB3: Breads containing fine (FB) or coarse (CB) buckwheat hull particles at an addition level of 3%. FB6/CB6: Breads containing fine (FB) or coarse (CB) buckwheat hull particles at an addition level of 6%.

The effect of BH particle size, BH addition level and the interaction between the two factors were significant (*p* < 0.05) for all crumb characteristics of the GFBs tested, except for uniformity and cell density for the BH particle size factor (Table 5 and Figure 2). With the exception of the FB6 sample, all BHBs showed a significant (*p* < 0.05) decrease in mean cell area, with the decrease ranging from 33% to 43%. In contrast to this parameter, an opposite trend was found for cell density, where all BHBs showed a significant (*p* < 0.05) increase compared to the control. The void fraction, which indicates the percentage of the cross-sectional area of the crumb that holds the gas cells, showed a significant (*p* < 0.05) decrease in BHBs compared to the control, except for the FB6 sample. These results were consistent with those obtained by Martínez et al. [42].

Crust and crumb colour parameters (L*, a*, b*, C*, h, ΔE) of BHBs are shown in Table 6. Both investigated factors (BH addition level and particle size) had a significant (*p* < 0.01) effect on the crumb luminosity (L*), whereas only the BH addition level had a significant (*p* < 0.05) effect on the same parameter of the crust. All BHBs showed significantly (*p* < 0.05) lower values than the control bread for crust and crumb luminosity and chromaticity (C*), as could also be noted from the colour difference (ΔE). Significantly (*p* < 0.05) lower crust luminosity was observed with increasing BH addition. In turn, the FBH addition induced a significant (*p* < 0.05) darkening effect with respect to the use of the coarse BH fraction. Lower L* values with fibre addition in bread have already been reported [32,42,45]. However, a darker GFB may not be perceived as negative, in contrast to the GFB commonly seen in market, characterised by a lighter colour than wheat bread [32].The effect of the studied factors in the crust and crumb colour coordinates (a* and b*) was significant (*p* < 0.05). Significantly (*p* < 0.05) lower a* and b* values were found for the crust of all BHBs compared to the control. Furthermore, the addition of CBH promoted the reduction of a* values more than the addition of FBH, as did the use of a 6% addition level compared to 3%. As crust chroma values are mainly determined by the Maillard reaction, the addition of the higher levels of fibres could have hindered this reaction. Anil demonstrated that breads with a lower dosage of hazelnut testa particles were more influenced by Maillard and caramelisation reactions than those with the higher dosage [13]. The a* coordinate of the crumb was significantly (*p* < 0.05) higher in all BHBs than in the control. The addition of FBH resulted in a more reddish crumb colour than the coarse BH. Since the temperature of the crumb is not as high as in the crust, the crumb colour is mainly related to the colour of the ingredients [25]. Therefore, the native BH colour could have determined the colorimetric characteristics of the crumb.

### 3.6. Sensory Evaluation

**Figure 3:**
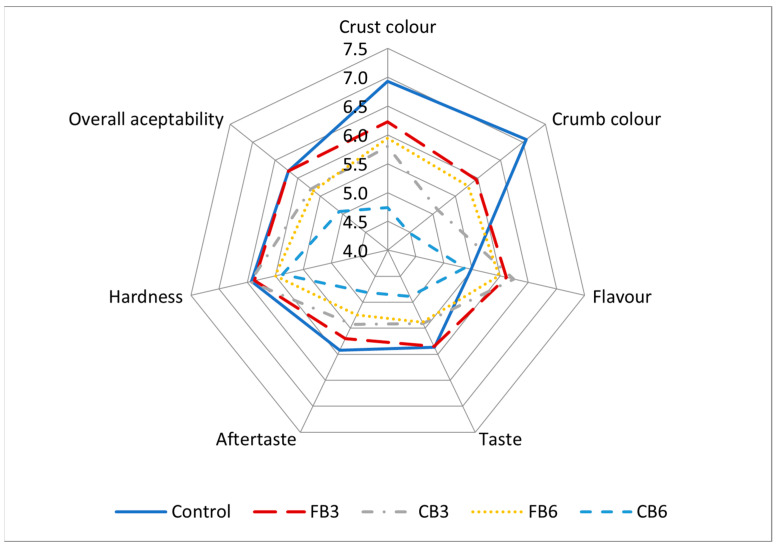
Effect of the addition of buckwheat hulls on the sensory properties of gluten-free breads. C: Control bread. FB3/CB3: Breads containing fine (FB) or coarse (CB) buckwheat hull particles at an addition level of 3%. FB6/CB6: Breads containing fine (FB) or coarse (CB) buckwheat hull particles at an addition level of 6%.

Figure 3 and Table S1 (supplementary material) show the results of the sensory attributes of GFBs that were evaluated by consumers. With the exception of the CB6 sample, consumers rated all the sensory attributes studied higher than five, with small variations between the different types of bread. Overall, consumers scored FBs better than CBs and the lower addition level better than the higher one. The effect of FB particle size was significant (*p* < 0.01) on crust and crumb colour. For these parameters, the panellist considered the crust and crumb colour of the control bread to be more appealing. In contrast, the flavour of the BHBs (except for the CB6 sample) was rated better than that of the control bread. As the addition level of BH particles was low, the characteristic bitter taste and aromatic intensity of BH [46] were not perceived as unpleasant by the panellists. Torbica et al. [46] observed that rice-based breads with no more than 10% inclusion of unhulled BW flour resulted in higher scores than breads containing dehulled BH flour. Panellists also gave similar scores to both kinds of FB samples, CB3 and the control bread in the following attributes: taste, aftertaste and hardness. However, CB6 samples showed a significant (*p* < 0.05) decrease in taste, aftertaste and overall acceptability, indicating that at this level of addition and using BH at this particle size, the GF bread resulted in impaired sensory attributes. On the other hand, no significant differences were found between the control and FB3 samples in terms of overall acceptability, proving that the FB3 samples had sensory attributes very similar to those of the control GF bread. Sabanis et al. [32] also reported higher scores for GF breads enriched with 3% cereal fibre added than those enriched with higher levels.

## 4. Conclusions

The use of BH as a food ingredient proved to be a very interesting ingredient to increase the fibre content of gluten-free breads. Its inclusion in the bread formulation also improved the total phenolic content and the antioxidant activity, especially in the breads formulated with the lowest particle size. These results were associated with a better extractability of compounds present in the hull as a consequence of the milling process. The increased antioxidant potential observed in the products formulated may be reflected in health-related properties; this possibility could be explored in future research studies through in vitro methods, such as simulated digestions and/or in vivo animal and clinical trials in order to explore the bioaccessibility and bioavailability of the bioactive compounds present in the BH. The bread samples formulated with higher BH concentrations showed a higher crumb hardness and a lower specific volume. The impairment of some bread quality parameters was related to the inclusion of a higher volume of BH particles, especially the larger ones, which could have hindered the formation of a proper crumb structure. The use of the lowest BH particle size mitigated the negative effect promoted by BH on the morpho-geometric properties and texture of the bread. Nevertheless, the bread obtained with the 3% FBH addition had a high fibre content and showed no adverse effects on bread quality properties compared to other BHB formulations. Furthermore, this bread was the best rated by consumers, receiving a score similar to that of the control sample. Therefore, the use of FBH as a low-cost source of fibre and bioactive compounds to enrich GF breads seems to be a good strategy to improve the nutritional content of GFBs without compromising the sensory properties of the breads. The same approach could be applied for the production of other fibre-rich, gluten-free bakery products.

## Figures and Tables

**Table 1:** Rheological properties of gluten-free doughs.

Sample	G′_1_ (Pa)	a	G″_1_ (Pa)	b	(tan δ) 1	c	G*_1_ (Pa)	Crosspoint (G′ = G″) (Pa)	τ_max_ (Pa)
C	712 ^a^	0.326 ^d^	400 ^a^	0.384 ^c^	0.561 ^d^	0.058 ^a^	817 ^a^	24 ^a^	1.67 ^a^
FB3	1480 ^c^	0.274 ^b^	682 ^c^	0.364 ^b^	0.462 ^b^	0.090 ^b^	1629 ^c^	42 ^c^	2.07 ^a^
CB3	1027 ^b^	0.299 ^c^	509 ^b^	0.384 ^c^	0.495 ^c^	0.086 ^b^	1147 ^b^	35 ^b^	1.85 ^a^
FB6	3738 ^e^	0.211 ^a^	1385 ^e^	0.307 ^a^	0.37 ^a^	0.096 ^b^	3985 ^e^	83 ^e^	3.25 ^b^
CB6	1973 ^d^	0.272 ^b^	896 ^d^	0.359 ^b^	0.454 ^b^	0.087 ^b^	2166 ^d^	50 ^d^	2.19 ^ab^
SE	76	0.003	28	0.004	0.004	0.003	57	1	0.86
Analysis of variance and significance of factors (*p*-values)									
BH particle size	**	**	**	**	**	ns	**	**	ns
BH addition level	**	**	**	**	**	ns	**	**	ns
Particle size × Addition level	**	**	**	**	**	ns	**	**	ns

C: Control dough. FB3/CB3: Doughs containing fine (FB) or coarse (CB) buckwheat hull particles at an addition level of 3%. FB6/CB6: Doughs containing fine (FB) or coarse (CB) buckwheat hull particles at an addition level of 6%. G′_1_ and G″_1_ represent the elastic and viscous coefficients at a frequency of 1 Hz after fitting to the power law model. G*_1_ refers to the complex modulus. τ_max_ represents the maximum stress in viscoelastic lineal region (LVR) obtained from the strain sweep. The crosspoint value represents the stress at G′ = G″. SE: Pooled standard error obtained from ANOVA. Values in the same column with different small letters are significantly different (*p* < 0.05). ** *p* < 0.01, * *p* < 0.05, ns: not significant.

**Table 2:** Proximal composition of gluten-free breads.

Sample	Carbohydrates (dw)	T.D.F. (dw)	Protein (dw)	Ash (dw)	Fats (dw)
	(%)	(%)	(%)	(%)	(%)
C	85 ^b^	2.8 ^a^	9 ^a^	1.9 ^a^	4.4 ^a^
FB3	84 ^ab^	8.0 ^b^	9 ^a^	1.9 ^a^	5.7 ^a^
CB3	84 ^ab^	8.5 ^b^	9 ^a^	1.9 ^a^	5.7 ^a^
FB6	83 ^ab^	13.1 ^c^	11 ^a^	1.9 ^a^	4.7 ^a^
CB6	81 ^a^	12.5 ^c^	13 ^a^	2.1 ^a^	4.5 ^a^
SE	1	0.4	1	0.1	0.5
Analysis of variance and significance of factors (*p*-values)					
BH particle size	ns	ns	ns	ns	ns
BH addition level	ns	**	ns	ns	ns
Particle size × Addition level	ns	ns	ns	ns	ns

C: Control bread. FB3/CB3: Breads containing fine (FB) or coarse (CB) buckwheat hull particles at an addition level of 3%. FB6/CB6: Breads containing fine (FB) or coarse (CB) buckwheat hull particles at an addition level of 6%. TDF: Total dietary fibre. Protein content was measured as N content × 6.25. SE: Pooled standard error obtained from ANOVA. Values in the same column with different small letters are significantly different (*p* < 0.05). ** *p* < 0.01, * *p* < 0.05, ns: not significant, dw: dry weight basis.

**Table 3:** Total phenol content and total antioxidant capacity of gluten-free breads.

Sample	TPC (mg GAE/ 100 g dw)	DPPH (mg TE/ 100 g dw)	ABTS (mg TE/ 100 g dw)	ORAC (mg TE/ 100 g dw)	FRAP (mmol Fe^2+^/ 100 g dw)	Q-DPPH (mg TE/ 100 g dw)	Q-ABTS (mg TE/ 100 g dw)
C	n.d.	19 ^a^	162 ^a^	78 ^a^	13.3 ^a^	2 ^a^	287 ^a^
FB3	0.6 ^a^	40 ^b^	191 ^ab^	135 ^b^	13.5 ^ab^	24 ^bc^	966 ^cd^
CB3	0.3 ^a^	38 ^b^	235 ^bc^	135 ^b^	13.8 ^b^	17 ^b^	878 ^bc^
FB6	2.1 ^b^	54 ^c^	279 ^c^	290 ^d^	15.1 ^d^	52 ^d^	1058 ^d^
CB6	1.8 ^b^	60 ^c^	261 ^c^	222 ^c^	14.6 ^c^	30 ^c^	799 ^b^
SE	0.3	3	20	4	0.2	3	43
Analysis of variance and significance of factors (*p*-values)							
BH particle size	ns	ns	ns	**	ns	**	**
BH addition level	**	**	*	**	**	**	ns
Particle size × Addition level	ns	ns	ns	**	**	*	ns

C: Control bread. FB3/CB3: Breads containing fine (FB) or coarse (CB) buckwheat hull particles at an addition level of 3%. FB6/CB6: Breads containing fine (FB) or coarse (CB) buckwheat hull particles at an addition level of 6%. TPC: Total phenol content. DPPH and Q-DPPH: Antioxidant capacity against the DPPH* radical in sample extracts and in solid samples, respectively. ABTS and Q-ABTS: Antioxidant capacity against the ABTS*+ radical in sample extracts and in solid samples, respectively. ORAC: Oxygen radical absorbance capacity of sample extracts. FRAP: Ferric reducing antioxidant power of sample extracts. SE: Pooled standard error obtained from ANOVA. Values in the same column with different small letters are significantly different (*p* < 0.05). ** *p* < 0.01, * *p* < 0.05, ns: not significant, n.d.: not detected, dw: dry weight basis.

**Table 4:** Bread quality properties of gluten-free breads.

Sample	Volume (mL)	Specific Volume (mL/g)	Weight Loss (%)	Hardness	Hardness (Day 7)	Chewiness	Resilience	Cohesiveness	Springiness
C	457 ^c^	3.0 ^b^	22.8 ^d^	1.8 ^a^	4.63 ^ab^	0.7 ^ab^	0.19 ^b^	0.45 ^bc^	0.85 ^a^
FB3	470 ^c^	2.8 ^b^	17.1 ^a^	2.2 ^a^	3.88 ^a^	0.8 ^ab^	0.21 ^c^	0.47 ^c^	0.80 ^a^
CB3	396 ^a^	2.5 ^a^	19.1 ^bc^	3.2 ^b^	6.81 ^d^	1.1 ^b^	0.17 ^a^	0.41 ^a^	0.81 ^a^
FB6	460 ^c^	2.8 ^b^	18.6 ^b^	3.0 ^b^	5.65 ^bc^	1.1 ^b^	0.18 ^ab^	0.43 ^ab^	0.82 ^a^
CB6	418 ^b^	2.6 ^a^	19.8 ^b^	3.1 ^b^	6.53 ^cd^	1.1 ^b^	0.17 ^ab^	0.41 ^a^	0.81 ^a^
SE	6	0.2	1.8	0.7	0.31	0.2	0.02	0.03	0.04
Analysis of variance and significance of factors (*p*-values)									
BH particle size	**	**	*	ns	**	ns	**	**	ns
BH addition level	ns	ns	**	**	**	**	ns	*	*
Particle size × Addition level	*	ns	ns	ns	**	ns	**	*	ns

C: Control bread. FB3/CB3: Breads containing fine (FB) or coarse (CB) buckwheat hull particles at an addition level of 3%. FB6/CB6: Breads containing fine (FB) or coarse (CB) buckwheat hull particles at an addition level of 6%. SE: Pooled standard error obtained from ANOVA. Values in the same column with different small letters are significantly different (*p* < 0.05). ** *p* < 0.01, * *p* < 0.05, ns: not significant.

**Table 5:** Crumb grain characteristics of gluten-free breads.

Sample	Mean Cell Area	Crumb Grain Uniformity	Cell Density	Void Fraction
	(mm^2^)		(cells/cm^2^)	(%)
C	0.30 ^b^	7 ^a^	44 ^a^	13.7 ^d^
FB3	0.19 ^a^	19 ^ab^	52 ^c^	10.0 ^b^
CB3	0.17 ^a^	35 ^b^	48 ^b^	8.3 ^a^
FB6	0.29 ^b^	8 ^a^	50 ^b^	15.7 ^e^
CB6	0.20 ^a^	14 ^a^	56 ^d^	11.6 ^c^
SE	0.01	5	1	0.4
Analysis of variance and significance of factors (*p*-values)				
BH particle size	**	ns	ns	**
BH addition level	**	*	**	**
Particle size × Addition level	**	ns	**	**

C: Control bread. FB3/CB3: Breads containing fine (FB) or coarse (CB) buckwheat hull particles at an addition level of 3%. FB6/CB6: Breads containing fine (FB) or coarse (CB) buckwheat hull particles at an addition level of 6%. SE: Pooled standard error obtained from ANOVA. Values in the same column with different small letters are significantly different (*p* < 0.05). ** *p* < 0.01, * *p* < 0.05, ns: not significant.

**Table 6:** Crumb and crust colour parameters of gluten-free breads.

Sample	Crust Colour						Crumb Colour					
	L*	a*	b*	C*	h	ΔE	L*	a*	b*	C*	h	ΔE
C	61 ^c^	13.3 ^e^	23 ^d^	27 ^e^	60 ^e^		74 ^e^	−0.5 ^a^	5.4 ^b^	5 ^a^	95 ^c^	
FB3	49 ^b^	10.8 ^d^	11 ^c^	15 ^d^	45 ^c^	17.0 ^a^	50 ^c^	8.3 ^d^	10 ^d^	13 ^d^	50 ^b^	26 ^b^
CB3	49 ^b^	7.8 ^bc^	10 ^b^	12 ^c^	51 ^d^	18.9 ^b^	53 ^d^	4.5 ^b^	5.8 ^b^	7 ^b^	52 ^b^	22 ^a^
FB6	44. ^a^	9.8 ^c^	5 ^a^	11 ^b^	26 ^a^	24.7 ^c^	42 ^a^	8.9 ^e^	6.6 ^c^	11 ^c^	35 ^a^	33 ^d^
CB6	45 ^a^	7.1 ^a^	5 ^a^	8 ^a^	33 ^b^	25.3 ^c^	45 ^b^	5.8 ^c^	4.0 ^a^	7 ^b^	37 ^a^	29 ^c^
SE	1	0.2	1	1	1	0.3	1	0.1	0.3	1	2	1
Analysis of variance and significance of factors (*p*-values)												
BH particle size	ns	**	*	**	**	*	**	**	**	**	ns	**
BH addition level	**	**	**	**	**	**	**	**	**	**	**	**
Particle size × Addition level	ns	ns	*	ns	ns	ns	ns	**	**	**	ns	ns

C: Control bread. FB3/CB3: Breads containing fine (FB) or coarse (CB) buckwheat hull particles at an addition level of 3%. FB6/CB6: Breads containing fine (FB) or coarse (CB) buckwheat hull particles at an addition level of 6%. L*, a*, b*: CIELAB colour coordinates, C*: chroma, h: hue, ΔE: difference of colour of each BHB sample with the control. SE: Pooled standard error obtained from ANOVA. Values in the same column with different small letters are significantly different (*p* < 0.05). ** *p* < 0.01, * *p* < 0.05, ns: not significant.

## Data Availability

The data presented in this research are available on request from the corresponding author.

## Supplementary Materials

The following supporting information can be downloaded at: https://www.mdpi.com/article/10.3390/foods12142781/s1, Table S1: sensory properties of gluten-free breads.
