# Supplementary material for: Valorisation of Buckwheat By-Product as a Health-Promoting Ingredient Rich in Fibre for the Formulation of Gluten-Free Bread
Source: Foods. 2023 Jul 21;12(14):2781. doi: 10.3390/foods12142781 (PMC10379109; doi:10.3390/foods12142781)
Supplement: Supplementary file 1 [file foods-12-02781-s001.zip › foods-2510346-supplementary.pdf]

Table S1. Sensory properties of gluten-free breads

Sample	Crust colour	Crumb colour	Flavour	Taste	Aftertaste	Crumb Texture	Overall acceptability
C	6.9 ^{c}	7.1 ^{d}	5.5 ^{ab}	5.9 ^{b}	5.9 ^{c}	6.43 ^{ab}	6.20 ^{c}
FB3	6.2 ^{b}	6.0 ^{c}	6.1 ^{bc}	5.9 ^{b}	5.7 ^{bc}	6.37 ^{ab}	6.20 ^{c}
CB3	5.8 ^{b}	5.1 ^{b}	6.2 ^{c}	5.4 ^{ab}	5.4 ^{abc}	6.44 ^{b}	5.70 ^{bc}
FB6	5.9 ^{b}	5.8 ^{c}	6.0 ^{abc}	5.4 ^{ab}	5.2 ^{ab}	6.00 ^{ab}	5.65 ^{b}
CB6	4.7 ^{a}	4.5 ^{a}	5.4 ^{a}	4.9 ^{a}	4.8 ^{a}	5.87 ^{a}	5.07 ^{a}
SE	0.2	0.2	0.2	0.2	0.2	0.20	0.19
Analysis of variance and significance of factors (*p-values*)							
BH particle size	**	**	ns	*	ns	ns	**
BH addition level	**	ns	*	*	*	*	**
Particle size x Addition level	ns	ns	ns	ns	ns	ns	ns

C: Control bread. FB3/CB3: breadss containing fine (FB) or coarse (CB) buckwheat hull particles at an addition level of 3%. FB6/CB6: breads containing fine (FB) or coarse (CB) buckwheat hull particles at an addition level of 6%. SE: pooled standard error obtained from ANOVA. Values in the same column with different small letters are significantly different ($p<0.05$). **$p < 0.01$, *$p < 0.05$, ns: not significant).
